# Wound Care During the COVID-19 Emergency in Padua Hospital, Italy

**DOI:** 10.1017/dmp.2020.448

**Published:** 2020-11-19

**Authors:** Giampiero Avruscio, Angelo Adamo, Chiara Tonello, Elisabetta Baracco, Fabiana Nalin, Ornella Scarpazzo, Lorenza Cacco, Sonia Ragazzo

**Affiliations:** 1 Angiology Unit, Department of Cardiac, Thoracic and Vascular Sciences, Hospital-University of Padua, Padua, Italy; 2 Department of Neuroscience, Hospital-University of Padua, Padua, Italy

**Keywords:** angiology, coronavirus, health care, pandemic, wound care

## Abstract

Chronic vascular wounds have a significant economic and social impact on our society, calling for the allocation of a great deal of attention and resources. The coronavirus disease (COVID-19) outbreak has represented a difficult challenge to face for health care providers and fragile patients, such as for outpatients and Day-Hospital patients needing continuous care at the Angiology Unit of the University Hospital of Padova in Italy, one of the most crucial areas worldwide. The project consisted of a critical revision of all the procedures from the patients’ arrivals to their discharge after completing the entire course of treatment. The previous standard of practice was modified according to the current evidence-based guidelines and the national and local government’s indications. The new standard of practice allowed our unit to protect every patient and staff member from the coronavirus infection, providing the same high standard of care as before the COVID-19 outbreak.

## Introduction

Vascular ulcers, especially of venous origin, are a relatively common condition in the adult population (1% of the population and 3.6% of people older than 65 years in developed countries) and are associated with significant morbidity, high cost of health care, loss of productivity, and reduced quality of life.^1–3^ Treatment can be expensive, leading to a large economic burden on health services: The annual cost for chronic venous lesions is estimated to be >$1 billion in the United States and between 400 and 600 million pounds in the UK.^1,4,5^ In the Province of Padova (Veneto region of Italy), the annual expenditure for 1421 patients receiving community health assistance is close to UK €6 million (US $6 402 060) (mean cost per patient UK €4225), and this is around 1% of the health care budget of the province (Ranzi M, unpublished data set, 2005).^4^

Coronavirus disease (COVID-19) is a viral disease caused by severe acute respiratory syndrome coronavirus 2 (SARS-CoV2) and was declared a pandemic by the head of the World Health Organization (WHO) and considered a public health emergency of international relevance.

Recommended preventive measures include hand washing, covering one’s mouth and nose when coughing or sneezing, maintaining distance from other people, wearing a face mask in public settings, and monitoring and self-isolating for people who suspect they are infected.^5^ Authorities worldwide have responded by implementing travel restrictions, lockdowns, workplace hazard controls, and facility closures. Many places have also worked to increase testing capacity and trace contacts of infected persons.

The present paper is the qualitative descriptive report of how the Angiology Unit of the University Hospital of Padova was able to keep performing a high-level wound care assistance during the COVID-19 pandemic in one of the most affected areas worldwide. The project was designed in response to a reassessment of internal procedures, which highlighted a series of critical points in managing high patient inflow.

### Setting

The Angiology Unit provides vascular medicine service for the whole Province of Padova (catchment population of ~1 million inhabitants). The unit is the “hub” for vascular ulcers. All major assessments are undertaken at the unit and all emergencies for the whole province are sent there. An outpatient clinic and a ward referral service are provided. The wound clinic is open daily; 6–7 patients are seen on average every day.^4^

### Methodology

In order to allow continuous access for patients needing wound care and urgent vascular evaluation and care, the following operating rules were applied:

**Figure 1.**
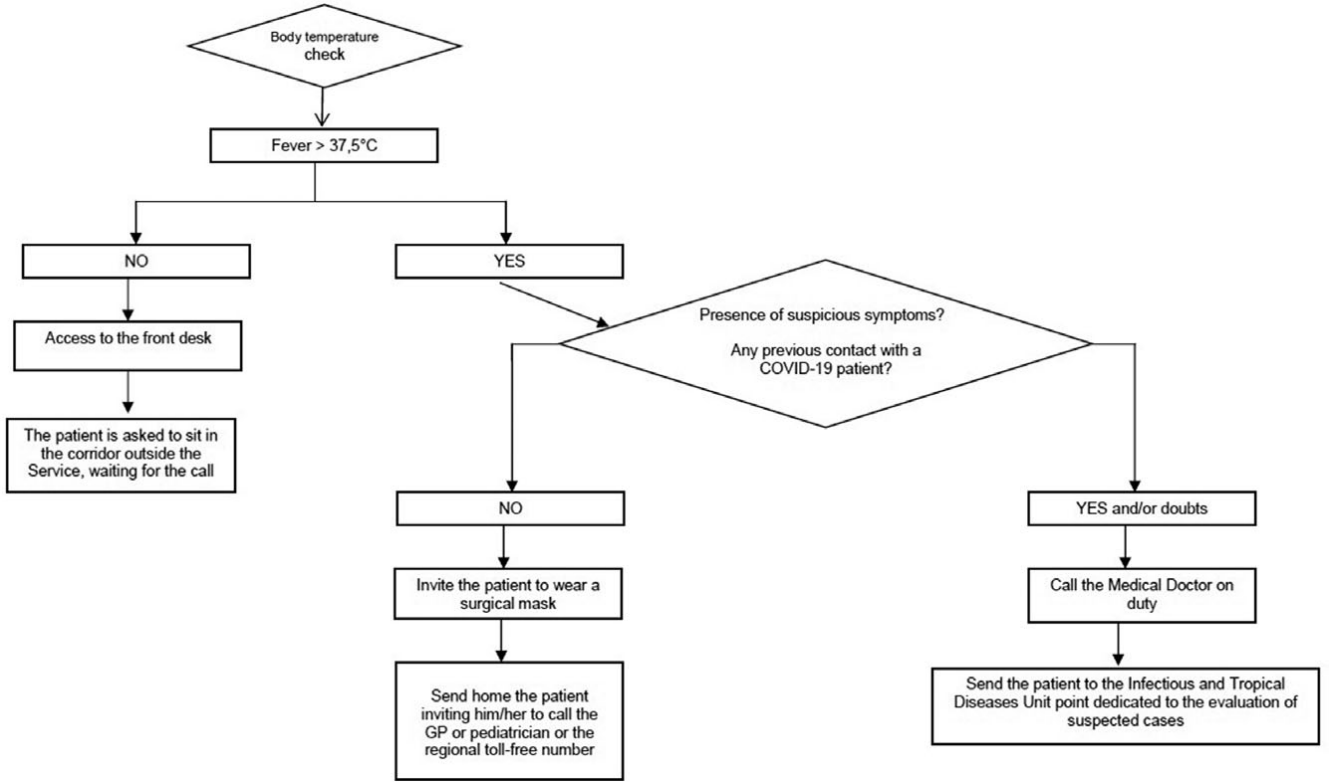
Flow chart for the security procedure to access the angiology unit.

#### Outpatients

1. Patients who accessed the Angiology Unit had to ring the bell and wait for the nurse on duty for the reception procedure. Information on the procedure to be followed was reported on information signs located near the unit entrance.
2. At the entrance door, a nurse equipped with the appropriate personal protective equipment (PPE) (FFP2 mask, gloves, and disposable gown) checked the body temperature using an infrared thermometer.
3. The nurse assessed whether the patient at the entrance was equipped with the PPE required; if not, the patient was provided with the necessary devices (eg, surgical mask even in the presence of an FFP3 mask with filter). Moreover, careful hand sanitation with alcoholic gel was implemented.

The medical and nursing staff of the Angiology Unit had to use the PPE required, regardless of the confirmed and/or doubtful positivity or negativity of COVID-19 or, in any case, in the presence of patients with febrile symptoms, cough, and/or flu-like syndrome.

4. The patient was given a questionnaire on his or her health condition, particularly focused on signs and symptoms typical of COVID-19 (ie, cough, frequently dry, asthenia, fatigue, dyspnea, sore throat, headache and muscle pain, chills, nasal congestion, runny nose, diarrhea for adults and the presence of the triad fever ≥ 37.5°C, dry cough, and unusual tiredness for pediatric patients), as required by the flow chart (Figure 1). Once admitted, the patient was asked to sit in the waiting area outside the service, recommending compliance with the safety distances of at least 1 meter (droplet-distance) between two persons.
5. In case the patient manifested a rise in temperature (≥ 37.5°C), suspected symptomatology, possible contact with people positive for COVID-19 in the last 14 days, doubtful answers, the nurse would have notified the medical doctor on duty, who would have then sent the patient to the Infectious and Tropical Diseases Unit, which is dedicated to the evaluation of suspected cases.
6. In the event of a rise in temperature, but low probability of a certain COVID-19 contact, the nurse would have notified the medical doctor on duty and sent the patient home inviting him/her to call the general practitioner or pediatrician or the regional toll-free number.
7. During the reception procedure, the nurse on duty had to limit the access of the patient’s caregiver, except for minors, disabled people, fragile patients, people who were not self-sufficient, or people with linguistic and cultural difficulties; in this case, only 1 accompanying person was allowed to enter, with the appropriate PPE.

If there was overcrowding in the waiting area outside the unit due to the presence of a large number of caregivers accompanying self-sufficient patients, they would be invited to wait elsewhere, such as in the cloister outside the hospital, in order to avoid gathering.

8. Once admitted, the patient underwent the scheduled examination during which he/she was invited to keep his/her head turned away from the operator, when possible, to avoid close face-to-face contact.
9. At the end of the procedure, the patient was invited to wait for the report in the waiting area outside the unit.
10. The nurse on duty, before admitting another patient, opened the windows to refresh the air and disinfected the examination table and all other surfaces that were in contact with the patient (chairs, table, etc) with 2.8% sodium hypochlorite and with special disposable alcohol-free disinfectant wipes (Cleanisept Wipes) for the ultrasound device and ultrasound probes.

#### Hospitalized Patients or Patients From the Emergency Department

1. Hospitalized patients or those admitted to the emergency department are already checked to be COVID-19-free in their respective wards, and they are accompanied to the Angiology Unit through specific protective routes.
2. After the vascular procedure, the patient was accompanied to the waiting room while the medical report was written and the return transport arrived. The nurse proceeded with the disinfection of the room and equipment according to the same procedures described previously.

#### Day Hospital

Before entering, the patients who accessed our Day Hospital had to carry out all the assessment procedures required as if they were outpatients. In addition, on the day of hospitalization, the patient had to undergo the nasopharyngeal swab for COVID-19 infection and wait in a separate room for the results before being admitted.

At the end of the infusion therapy cycle, after discharging the patient, the nurse had to disinfect the chair and other surfaces that were in contact with the patients.

## Results

No cases of COVID-19 infection have been reported among any of the medical and nursing staff of the Angiology Unit during the entire period of the maximum outbreak in Italy, as well as among the patients who accessed the unit. Moreover, the Angiology Unit was able to carry out all the wound care and urgent activities as practiced before the COVID-19 outbreak began.

## Discussion

The previous results are consistent with the 1.9% infection rate of the 8000 health care professionals of the University Hospital of Padova from the beginning of the outbreak in Italy on February 21 to the end of phase 1 emergency at the end of May (144 infected, most of whom were asymptomatic). This was due to a quick and efficient reorganization, with the implementation of separate COVID-19 and COVID-19-free paths and dedicated COVID-19 wards, as well as the active search for the asymptomatic carriers with a massive screening protocol (consisting of the largest number of COVID-19 tests performed respective to the worldwide population), despite the initial different indications provided by the WHO.

The study on the outbreak of COVID-19 in the Italian municipality of Vo’, in the Province of Padova, has in fact demonstrated that the viral load of the asymptomatic patients is the same as that of the symptomatic patients. This study sheds light on the frequency of asymptomatic COVID-19 infection and infectivity, and provides insights into the virus’s transmission dynamics and the efficacy of the implemented control measures.^6^

**Figure 2.**
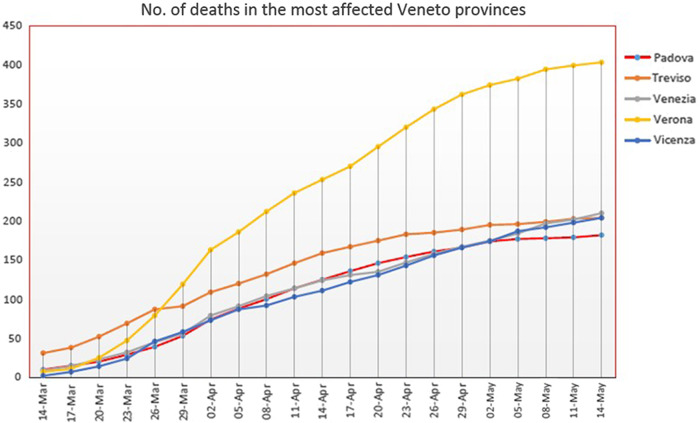
The graph shows the low mortality of Padua respect to other Veneto cities.

**Figure 3.**
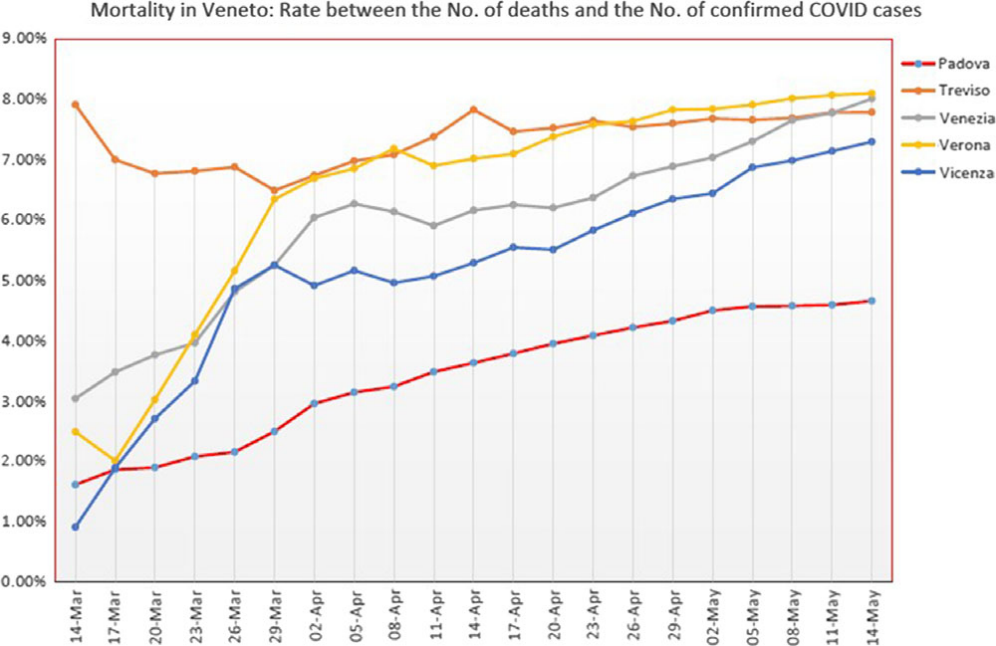
The graph shows the low lethality of Padua respect to other Veneto cities.

This study, along with an effective reorganization of the University Hospital of Padova and a careful health surveillance through periodic nasopharyngeal swabs, depending on the degree of risk of the health care professionals, has constituted a “model” that has resulted in Padova having the lowest lethality (intended as the ratio between the number of deaths and the number of those infected) and the lowest mortality in relation to other cities in the Veneto, as seen from the data reported by the regional government (see Figure 2 and Figure 3).

WHO’s guidance in “Non-pharmaceutical public health measures for mitigating the risk and impact of epidemic and pandemic influenza” conditionally recommends a face mask be used in the community for asymptomatic individuals in severe epidemics or pandemics in order to reduce transmission in the community; this is based on mechanistic plausibility for the potential effectiveness of this measure.^7^ Thus, we could assume that the use of at least a surgical mask by both the health care workers and the patients, together with the body temperature check and all the procedures described earlier, is an example of good clinical practice in providing continuous health care for patients, even in the presence of such a pandemic.

## Conclusion

Overall, this methodology report emphasized the importance of standardization of practice according to the latest health care safety guidelines to continue performing vascular wound care and urgent vascular evaluation and care during a pandemic, such as COVID-19 and the consequent lockdown.^8^ Fragile patients followed by our Angiology Unit for vascular ulcers or critical limb ischemia have been able to access proper cures, resulting in a sensible gain in terms of public health and costs. The new standard of practice allowed our unit to protect every patient and staff member from the new coronavirus infection, providing the same high standard of care as before the COVID-19 outbreak began.

Both the mentioned methodology scheme and the close collaboration with the scientific community have had distinguishing features from the beginning and pillars of the “Padova Model.”
